# Clinical efficacy of three plating systems in management of mandibular parasymphyseal fractures: a retrospective study

**DOI:** 10.1186/s12903-024-05143-3

**Published:** 2024-11-14

**Authors:** Nermine Ramadan Mahmoud, Yasser Fekry Habaka

**Affiliations:** Oral and maxillofacial surgery Department, Faculty of Dentistry, October 6th University, 6th of October City, Giza Governorate Egypt

**Keywords:** Parasymphyseal fracture, Miniplates, Maxillomandibular fixation

## Abstract

**Background:**

The main goal of management of the mandibular fracture is to obtain the pre-injury contour of the bone and pre-injury occlusion as well as regain function early. For Parasymphyseal fractures, the ideal fixation method is still debatable. It could range from rigid to semi-rigid fixation. The current study aimed to compare the clinical and radiographic effect of two miniplates, 2.3 high-profile Miniplates, and three-dimensional (3D) Miniplates for internal fixation treatment of mandibular para-symphyseal fractures.

**Methods:**

The study reviewed 240 medical records, dividing 60 patients into three treatment groups: Group I (25 patients received 2.0 mini plates), Group II (22 patients received 2.3 high profile miniplate), and Group III (13 patients received 3D miniplate). Data included demographics, fracture diagnosis, radiographs, maxillomandibular fixation use, surgical time, intervention, postoperative stability, and complications.

**Results:**

The present study included 60 patients with mandibular parasymphyseal fractures, divided into three treatment groups: Group I (*n* = 25, 41.67%), Group II (*n* = 22, 36.67%), and Group III (*n* = 13, 21.67%). The study found no significant differences in age and sex distribution among the three groups, but the mean surgery time varied significantly. Group II had the highest rate of complications at 22.73%, followed by Group I at 20% and Group III at 7.69%.

**Conclusion:**

In conclusion, the (3D) Miniplates could provide better internal fixation treatment of mandibular para-symphyseal fractures in terms of easy use, short surgery time and less complication such as malocclusion and infection.

## Introduction

Maxillofacial trauma has a significant impact on patients admitted to trauma centers in various regions of the world. This impact is primarily because the maxillofacial region is the most visible and least protected part of the body, affecting both function and aesthetics. Despite being the largest and strongest among the face bones, the mandible is the second most fractured bone after the nasal bone [1, 2]. Different studies have shown different fracture locations in the mandible. Nearly 35% of individuals had parasymphyseal fractures [2–6].

The treatment of fractures in the maxillofacial region has changed throughout the past decades. Management of mandibular fractures has progressed from bandage use or Gunning splint to interdental wiring, and open reduction with plates and screws. Different types of plating systems have been used to provide stable fixation for mandibular fractures and osteotomies. A wide variety of plates and screws have been used such as the bicortical plating system, miniplates, 2.3 high profile miniplate, lag screws, locking miniplates, resorbable plates, and 3-dimensional plates [7–12].

The main goal in managing mandibular fracture is to restore the pre-injury contour of the bone and pre-injury occlusion as well as regain function early. For parasymphyseal fractures, the ideal fixation method is still debatable. It could range from rigid to semi-rigid fixation. The concept of the “Ideal osteosynthesis line’’ has been mentioned by Champy’s for the semi-rigid fixation by using mono-cortical miniplates along the lines [11–13].

Champy’s semi-rigid fixation approach employed the two bendable monocortical miniplates along an optimum osteosynthesis line. Plates arranged along these lines were supposed to provide the most functionally effective fixing and stability. Some surgeons recommend IMF for 1–2 weeks after fixation of the fracture using a miniplate due to torsional movements under function that may result from their small size and reduced rigidity [12–14].

To overcome the disadvantages of the miniplate, a low-profile, high mechanical-strength 2.3 miniplate system was introduced. This plate system can withstand both tension and compression pressures [12, 13, 15]. However, it had been hard to balance between adequate rigid internal fixation and bony and soft tissue preservation for effective bone healing. Many studies showed soft tissue dehiscence with high-profile plates, especially in the anterior region of the mandible [13, 16].

The shortcomings in the plating system used in the management of mandibular fracture led to the development of three-dimensional plates. The stability of the plate is driven from its design rather than thickness of the hardware. The clinical studies with three dimensional plates reported low complication rates and concluded that the 3-Dimensional plates used are the alternatives to conventional manipulates for the treatment of mandibular fracture. Moreover, it is easy to manipulate intraoperative and easily adapted over the bone provide stability for both tension and compression zones across the fracture segment [17–19].

The current literature compares the clinical outcomes of miniplates and reconstruction plates has not demonstrated any statistically significant differences in complication rates. However, the selection between these two fixation methods often depends on the complexity of the fracture pattern and the anticipated biomechanical requirements of the repair. Moreover, evidence on the use of three-dimensional (3D) remains limited, the available evidence suggests potential advantages of this technology in facilitating improved bone healing and achieving a more patient-specific implant fit. Some studies compared those treatment modalities regarding the clinical outcomes [2, 18–20].

The current study aimed to compare the clinical and radiographic effect of two miniplates, 2.3 high profile Miniplates, and three dimensional (3D) Miniplates for internal fixation treatment of mandibular para-symphyseal fractures.

## Material & methods

This is a retrospective study. All the patients with parasymphyseal fractures received open reduction and internal fixation (ORIF) Oral and Maxillofacial Surgery Department, Faculty of Dentistry, October 6 University were reviewed from January 2019 till December 2023. The study was approved by the Faculty of Dentistry Research Ethics Committee under approval no (RECO6U/18-2019) and the trial protocol was registered on clinical-trials.org (ClinicalTrials.gov ID: NCT06323122).

Upon this admission to the hospital, all patients involved in the study signed an informed consent form authorizing the use of their de-identified medical records for scientific research trials. The study was conducted according to the Helsinki Declaration and its later amendments.

Medical records were reviewed for all patients who underwent open reduction with internal fixation of Parasymphyseal fractures with the use of 2.0 miniplates, 2.3 high-profile miniplates, and 3D miniplates. The study inclusion criteria were; patients who underwent of ORIF parasymphyseal mandibular fracture, Patients with a sufficient number of healthy teeth (at least 4–6 teeth on each jaw) for MMF and patients who treated within 7 days of injury. The exclusion criteria included patients with condylar fracture, patients with comminated fractures, patients with associated bone pathology, patients with infection at the site of fracture, patients with loss of bone or soft tissue, cases lost to immediate follow-up following surgical procedures, and Pediatric patients with mixed dentition.

The primary predictor in the study was the plating technique. The primary outcome measures were the surgical time, and the secondary outcome was the fracture stability and complication associated with each technique.

A total of 240 medical records were reviewed and 60 patients met the inclusion and exclusion criteria, divided into three treatment groups: Group I; patients received 2.0 mini plates (*n* = 25, 41.67%), Group II; patients received 2.3 high profile miniplate (*n* = 22, 36.67%), and Group III; patients received 3D miniplate (*n* = 13, 21.67%).

### Surgical procedures

**Fig. 1:**
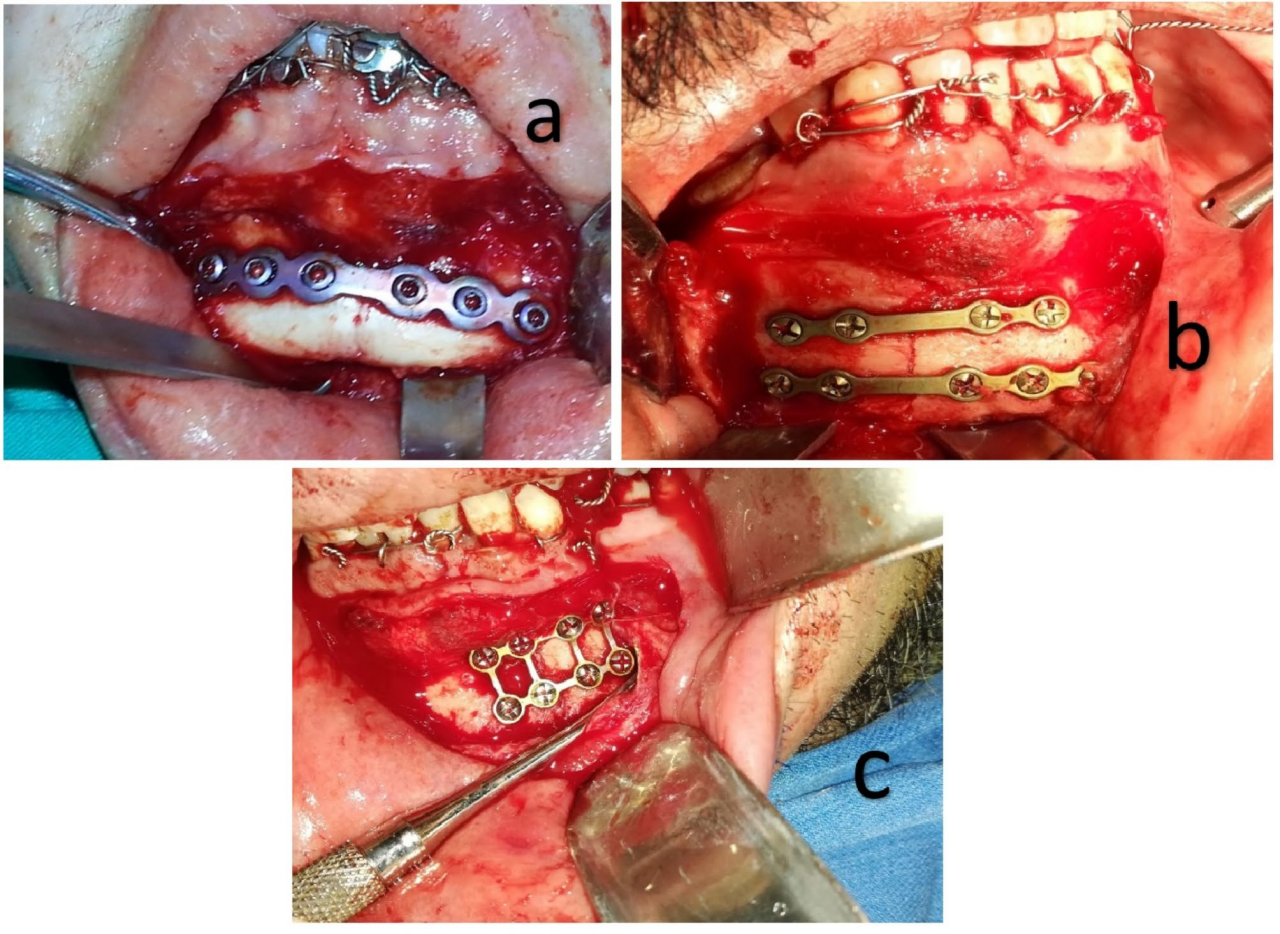
Fixation of plates system along the fracture line. **a** group I. **b** group II. **c** group III

Under general anesthesia and nasotracheal intubation, the procedure was carried out. First, the oral cavity was cleaned with povidone-iodine. Standard surgical procedures were revised for all patients. Maxillary and mandibular arch bars were applied and secured with 24-gauge circumferential wire. The fracture was reduced by establishing centric occlusion and placing IMF wires. Before the incision, the site was infiltrated with local anesthesia containing 4% articaine hydrochloride with 1:1,00,000 epinephrine (ARTINIBSA 40 mg/mL + 0.01 mg/mL solution for injection, Inibsa). The fracture site was exposed intraorally. A vestibular, curvilinear incision was placed through the mucosa, 5–8 mm from the mucogingival junction. After incising the mentalis muscle, subperiosteal dissection and skeletonization of the nerve was carried out to expose the fracture segments. In group I, a double 4-hole non-compression titanium miniplate was adapted at the superior and inferior borders of fracture. Drilling was done with the help of a 1.5-mm SS drill bit under copious saline irrigation. Conventional miniplates were fixed by inserting self-tapping screws (7 mm in length and 2 mm in diameter) through the holes using screw-drivers). In group II, a reconstruction plate was selected and adapted with plate benders. Drilling was done with the help of a 1.5-mm SS drill bit under copious saline irrigation. A reconstruction plate was fixed by inserting self-tapping screws (9 mm in length and 2 mm in diameter) through the holes using screw-drivers. In group III, a 3D miniplate was selected and adapted at the fracture line. Drilling was done with the help of a 1.5-mm SS drill bit under copious saline irrigation. Conventional miniplates were fixed by inserting self-tapping screws (7 mm in length and 2 mm in diameter) through the holes using screw-drivers). (Fig. 1)

**Fig. 2:**
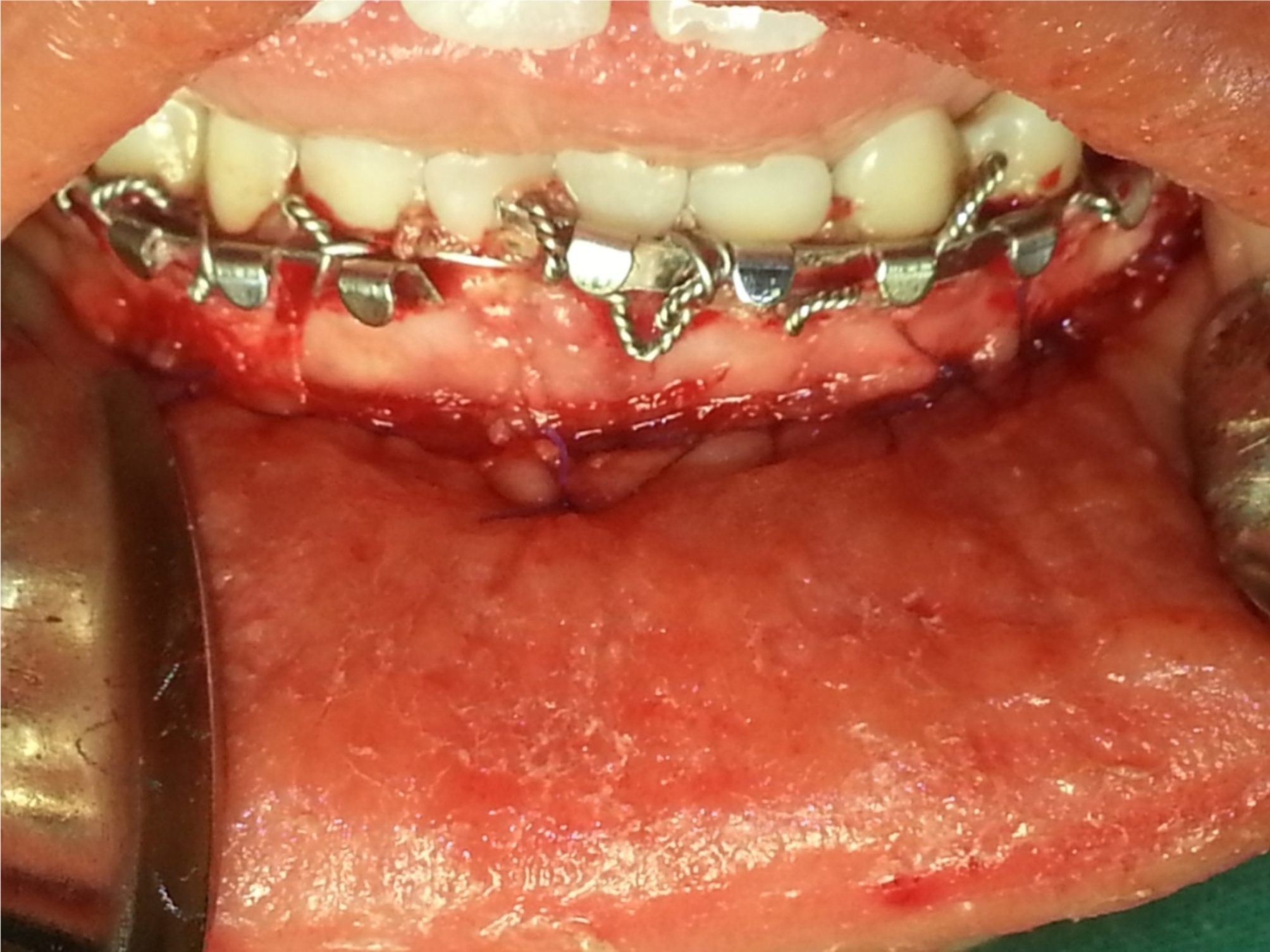
Surgical wound closure

After the required number of screws and plates was placed for securing fracture fragments, the surgical site was irrigated with normal saline. The incision was closed tightly. (Fig. 2).

The concomitant fractures fracture associated with parasymphyseal mandibular fracture were treated in a staged procedure through open reduction and internal fixation with miniplates and screws.

### Data collected & covariant

**Fig. 3:**
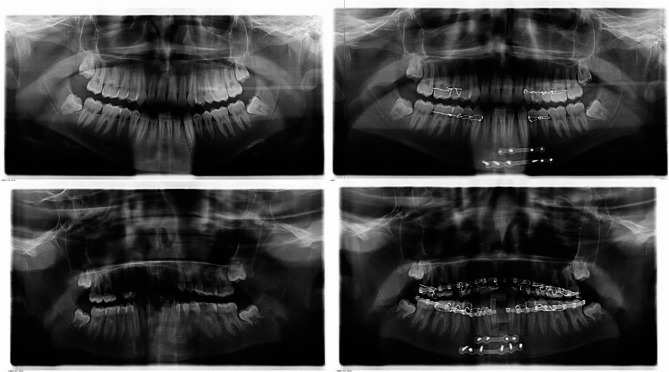
Pre& post-operative radiographic evaluation

The data collected from patients’ medical record to provide a detailed description of each patient data. the patient demographics; include basic information about the patients, such as age, sex, and any relevant medical history. The site and location of the fracture were determined by clinical and radiographic records.

radiographic records were evaluated to determine the number and location of fracture lines and the degree of displacement preoperative. In contrast, the postoperative radiograph records were evaluated to determine the reduction of the fracture by assessment of the inferior border of the mandible. Whenever alignment of the inferior border of the mandible was maintained across the fracture line, it was considered an excellent reduction. If a discrepancy of 1–3 mm was noted, it was considered a good reduction. If a discrepancy of 3–5 mm was noted, it was labeled as a fair reduction. If the discrepancy was > 5 mm, it was considered a poor reduction (Fig. 3) [21].

The surgical time was determined as the total time of operation starting from incision till suturing. It was timed in minutes. The postoperative fracture stability was evaluated based on occlusal derangement through the follow-up in the patient records. The stability was scored as follows Stable (all occlusal contacts are preset between maxillary and mandibular teeth on both sides); Mild derangement (a slight gap is present between one or two cusps on the unilateral or bilateral sides); Unstable and grossly deranged occlusion (no occlusal contacts between maxillary and mandibular teeth are present). postoperative complications, including nerve injury, plate exposure, wound infections, malunion, nonunion, and malocclusion were recorded as present or not.

The healing of concomitant fracture was recorded in the patients’ records and any complication that could impact the outcomes of the study was excluded from analysis.

### Statistical analysis

The statistical analysis was performed using R version 4.3.2 in conjunction with the tidyverse, psych, ggplot2, rstatix, and Stata packages. Categorical data were described using frequencies and percentages. The chi-square test or Monte Carlo test was employed to assess the association between the study groups and the variables of interest.

The normality of continuous data was evaluated using the Shapiro-Wilk test. Normally distributed data were summarized as mean ± standard deviation, while non-normally distributed data were presented as median (minimum-maximum). The corrected Welch’s ANOVA was utilized to examine the relationship between the study groups and surgery time and age, followed by Tukey’s post-hoc test for pairwise comparisons.

For binary categorical variables, the association with surgery time was analyzed using the t-test or Mann-Whitney U test, depending on the normality assumption. The Kruskal-Wallis test was employed for non-normally distributed data involving more than two groups. We also used Person correlation test. The level of statistical significance was set at *p* < 0.05 for all analyses.

## Results

The present study included 60 patients with mandibular parasymphyseal fractures, divided into three treatment groups: Group I (*n* = 25, 41.67%), Group II (*n* = 22, 36.67%), and Group III (*n* = 13, 21.67%). The mean age of the study population was 32.58 ± 8.15 years, with no statistically significant difference observed among the groups (*p* = 0.48). Male patients predominated across all groups, comprising 80% (*n* = 20) in Group I, 81.82% (*n* = 18) in Group II, and 61.54% (*n* = 8) in Group III, with no significant variation in sex distribution according to Table 1.

**Table 1:** Demographic and fracture characteristics among study groups (*N* = 60)

Demographics and fracture Characteristics		Group I 25(41.67%)	Group II 22(36.67%)	Group III 13(21.67%)	*p*
Sex					0.342
	Female	5 (20%)	4 (18.18%)	5 (38.46%)	
	Male	20 (80%)	18 (81.82%)	8 (61.54%)	
Age	Mean ± SD	32.96 ± 8.9	33.41 ± 8.2	30.46 ± 6.67	0.48 ^a^
Fracture cause					0.257 ^b^
	Fall	2 (8%)	1 (4.55%)	1 (7.69%)	
	IPV	3 (12%)	8 (36.36%)	2 (15.38%)	
	RTA	20 (80%)	13 (59.09%)	10 (76.92%)	
Associated fracture					0.217^b^
	angle	4 (16%)	2 (9.09%)	1 (7.69%)	
	body	7 (28%)	0 (0%)	3 (23.08%)	
	isolated	14 (56%)	17 (83.36%)	9 (69.23%)	
	Lefort I	0 (0%)	1 (4.55%)	0 (0%)	
	ZMC	0 (0%)	2 (9.09%)	0 (0%)	
Fracture side					0.524
	bilateral	12 (48%)	7 (31.82%)	5 (38.46%)	
	unilateral	13 (52%)	15 (68.18%)	8 (61.54%)	

a: T-test, b: Montecarlo test

Road traffic accidents (RTA) were the leading cause of fractures, accounting for 80% (*n* = 20) in Group I, 59.09% (*n* = 13) in Group II, and 76.92% (*n* = 10) in Group III. Interpersonal violence (IPV) was the second most common etiology, with 12% (*n* = 3) in Group I, 36.36% (*n* = 8) in Group II, and 15.38% (*n* = 2) in Group III. The distribution of fracture causes did not differ significantly among the groups.

Isolated Parasymphyseal fractures were the predominant pattern of associated fractures, observed in 56% (*n* = 14) of Group I, 83.36% (*n* = 17) of Group II, and 69.23% (*n* = 9) of Group III. No statistically significant difference was found in the distribution of associated fracture patterns among the groups.

Regarding the fracture side, unilateral fractures were more common in Group I (52%, *n* = 13) and Group III (61.54%, *n* = 8), while bilateral fractures predominated in Group II (68.18%, *n* = 15). However, the difference in fracture side distribution among the groups was not statistically significant.

**Fig. 4:**
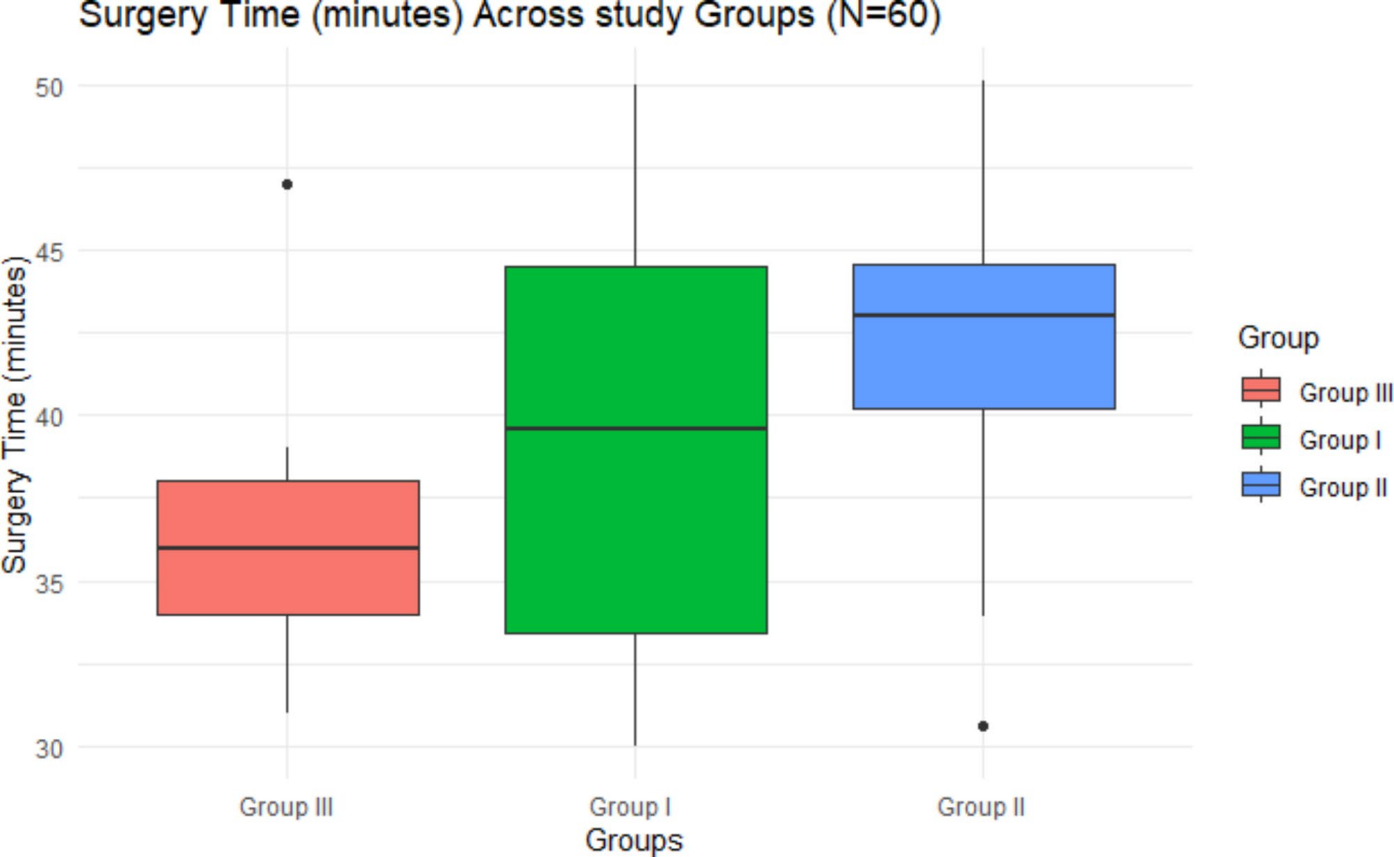
Surgery time (minutes) across study groups (*N* = 60)

As appeared in Table 2; Fig. 4, the mean surgery time differed significantly across the three groups (Group I: 38.43 ± 5.96 min, Group II: 42.43 ± 4.96 min, and Group III: 36.38 ± 4.09 min), as determined by Welch’s ANOVA due to the violation of the homogeneity of variances assumption, F (2, 34.69) = 7.78, *p* = 0.001. The effect size, calculated using eta-squared, was 0.82, indicating a large effect.

**Table 2:** Surgical outcomes and complications Across Treatment groups (*N* = 60)

surgical outcomes and complications		Group I 25(41.67%)	Group II 22(36.67%)	Group III 13(21.67%)	*p*
Surgery time		38.43 ± 5.96	42.43 ± 4.96	36.38 ± 4.09	0.001*^a^
Fracture stability	yes	24 (96%)	22 (100%)	12 (92.31%)	0.458
	No	1 (4%)	0 (0%)	1 (7.69%)	
Type of complication	infection	1 (4%)	1 (4.55%)	0 (0%)	0.835
	occlusion	3 (12%)	2 (9.09%)	0 (0%)	
	paresthesia	1 (4%)	2 (9.09%)	1 (7.69%)	
	no complication	20 (80%)	17 (77.27%)	12 (92.31%)	
Presence of complications	Yes	5 (20%)	5 (22.73%)	1 (7.69%)	0.519
	No	20 (80%)	17 (77.27%)	12 (92.31%)	

a: One way ANNOVA Welch test, * *p*-value : <0.05

Tukey’s post-hoc test revealed a statistically significant difference in mean surgery time between Group II (42.43 ± 4.96 min) and Group III (36.38 ± 4.09 min) (*p* < 0.05). However, no significant differences were found between Group I and Group II or between Group I and Group III.

Regarding fracture stability, most patients in all groups achieved satisfactory results, with 96% (*n* = 24) in Group I, 100% (*n* = 22) in Group II, and 92.31% (*n* = 12) in Group III experiencing stable fracture reduction. However, the difference in fracture stability rates among the groups did not reach statistical significance.

The distribution of complication types, including infection, occlusal discrepancies, and paresthesia, did not differ significantly among the groups (*p* = 0.835). The most common complication observed was occlusal disturbances, affecting 12% (*n* = 3) of patients in Group I and 9.09% (*n* = 2) in Group II. Notably, no instances of infection were reported in Group III.

Overall, the presence of complications did not vary significantly among the treatment groups. Group II exhibited the highest rate of complications at 22.73% (*n* = 5), followed by Group I at 20% (*n* = 5), and Group III at 7.69% (*n* = 1).

**Fig. 5:**
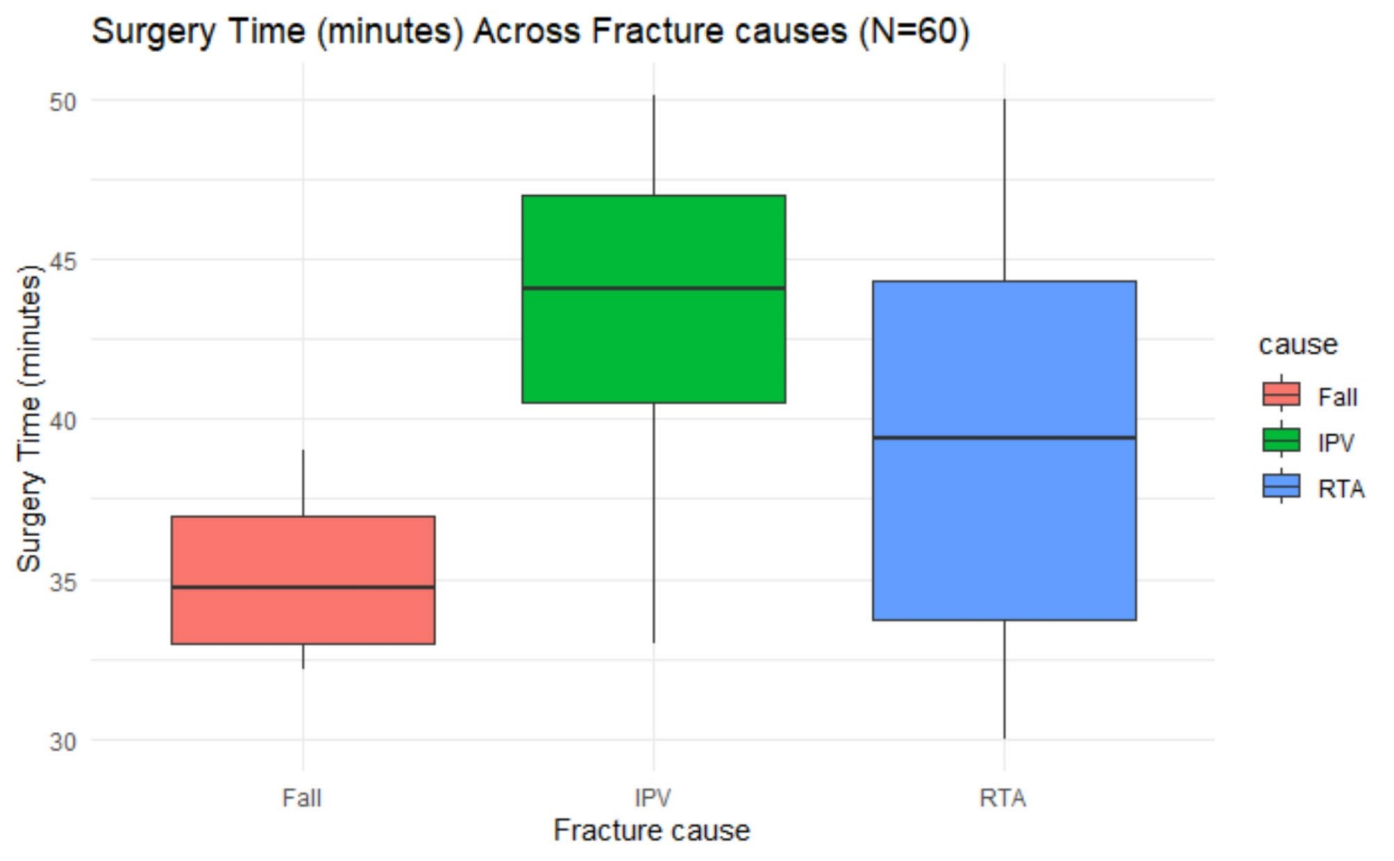
Surgery time (minute) vs. fracture causes (*N* = 60)

In our research, we’ve been examining the relationship between the duration of surgery and the nature of the fracture case. Our findings, which are detailed in Table 3; Fig. 5, reveal some significant associations.

**Table 3:** Comparative analysis of surgery time and study demographics, characteristics, and complications (*N* = 60)

Demographics, characteristics, complications		Surgery time	*p*
	Mean ± SD		
Age	32.58 ± 8.15	39.45 ± 5.7	0.915^a^
Sex	Female	39.69 ± 5.26	0.965^b^
	Male	39.38 ± 5.88	
Fracture cause	Fall	33.2(32.2–39)	0.014*^c^
	IPV	44.1(33-50.1)	
	RTA	39.4(30–50)	
Associated fracture	angle	39.4(34.4–46.3)	0.785
	body	35.8(30.4–50)	
	isolated	40.1(30-50.1)	
	Lefort I	40.5(40.5–40.5)	
	ZMC	42.45(40.5–44.4)	
Fractur Side	bilateral	38.1 ± 4.99	0.121^b^
	unilateral	40.35 ± 6.02	
fracture stability	yes	39.52 ± 5.78	0.382
	No	37.45 ± 2.19	
type of complication	infection	30.5(30.4–30.6)	0.108^c^
	occlusion	40.1(30-40.5)	
	paresthesia	43.95(32-44.6)	
	no complication	39.6(31-50.1)	
Presence of complications	Yes	37.5 ± 5.88	0.238^b^
	No	39.89 ± 5.62	

a: Person Correlation test, b: independent t test, c: Kruskal Wallis test, * *p*-value < 0.05

We observed that the median surgery time for falls was approximately 33.2 min, with an interquartile range (IQR) of 32.2–39 min. For cases related to Intimate Partner Violence (IPV), the median surgery time was slightly longer, around 44.1 min, with an IQR of 33–50.1 min. In the case of Road Traffic Accidents (RTA), the median surgery time was about 39.4 min, with an IQR of 30–50 min.

These differences in surgery times were found to be statistically significant, as indicated by a *P*-value of 0.014. Further analysis using Tukey’s post hoc test revealed a significant difference specifically between IPV and RTA cases.

However, our study did not find any significant association between the surgery time and patient demographics, complications, or other fracture characteristics.

## Discussion

Various plating techniques have been employed to offer stable fixation for mandibular fractures and osteotomies. The bicortical plating system, miniplates, 2.3 high profile miniplate, lag screws, locking miniplates, resorbable plates, and three-dimensional plates have all been employed [2–6].

The present study compared the effectiveness of two miniplates, 2.3 high-profile Miniplates, and 3D Miniplates, for internal fixation treatment of mandibular para-symphyseal fractures, evaluating fracture stability and postoperative complications.

In the current study, the parasymphyseal fracture was the main inclusion criteria as the parasymphyseal fracture represents the most common mandibular fracture. The frequency of parasymphyseal and symphyseal fractures has been reported to be 9–57% ^(1–3)^. This is mostly due to the lack of the supportive components observed in posterior tooth-bearing mandible fractures, interdigitation of molar and premolar teeth, and balancing generated by the lateral masseter and medial pterygoid muscles, which assemble the natural pterygomasseteric sling [20]. Moreover, the choice of the parasymphyseal fracture based on the complexity of anatomy of the mandible and vector of forces produced by different muscles. The forces act in a direction that separate the inferior borders at the site of fracture.

In the study, the Isolated Parasymphyseal fractures were the predominant pattern of associated fractures, observed in 56% (*n* = 14) of Group I, 83.36% (*n* = 17) of Group II, and 69.23% (*n* = 9) of Group III. This was in favor of the study setting to give a chance of evaluating the isolated parasymphyseal fracture. Moreover, the study excluded the condylar fracture as associated fracture in favor of avoiding the negative impact of condylar fracture on the postoperative occlusion.

Regarding fracture stability, most patients in all groups achieved satisfactory results, with 96% (*n* = 24) in Group I, 100% (*n* = 22) in Group II, and 92.31% (*n* = 12) in Group III experiencing stable fracture reduction. This indicates higher stability of the 2.3 high profile miniplate and the 3D- miniplate showed the least stability this could be explained as several patients were the lowest among the 3D- miniplate group. the high profile of the mini plate that was able to resist all the loads across the fracture line as well as achieved remarkable normalization of morphology with rigid inner fixing. It also provides good stability, allowing for extensive bone repair [7, 10, 22].

On the other hand, different studies reported that 3D- miniplate provide high stability across the fracture line. The high stability refers to their ability to provide stability in three dimensions, resist forces, and maintain a low profile and malleability. As well as The 3D plate’s quadrangular design distributes stresses across a large surface area rather than along a single line. This improves stability against torsional forces, vertical displacement, bending, and shearing forces [18, 23, 24].

The mean surgery time differed significantly across the three groups (Group I: 38.43 ± 5.96 min, Group II: 42.43 ± 4.96 min, and Group III: 36.38 ± 4.09 min). Tukey’s post-hoc test revealed a statistically significant difference in mean surgery time between Group II (42.43 ± 4.96 min) and Group III (36.38 ± 4.09 min). This indicates significant reduction in the surgery time in favor of 3D miniplate group. This is due to easily adaptation of the palate to the bone without deformation or displacement. Additionally, both the superior and inferior borders can be stabilized simultaneously [18, 23, 24]. This is in line with Al-Tairi et al. [25], who compared three-dimensional plate versus double-miniplate osteosynthesis for the stability of unfavorable mandibular and reported less surgery time to the 3D miniplate. Furthermore, our findings were consistent with those of Al-Moraissi and Ellis [26], who reported a substantial difference in operating time between 3D plates and 2 miniplates in the management of symphyseal and parasymphyseal fractures. Examining the relationship between the duration of surgery and the nature of the fracture case. the median surgery time for falls was approximately 33.2 min. while cases related to Intimate Partner Violence (IPV), the median surgery time was slightly longer, around 44.1 min and in the case of Road Traffic Accidents (RTA), the median surgery time was about 39.4 min.

The distribution of complication types, including infection, occlusal discrepancies, and paresthesia, did not differ significantly among the groups (*p* = 0.835). The most common complication observed was occlusal disturbances, affecting 12% (*n* = 3) of patients in Group I and 9.09% (*n* = 2) in Group II. Notably, no instances of infection were reported in Group III. Overall, the presence of complications did not vary significantly among the treatment groups.

In the current study, satisfactory occlusion was achieved in all cases of 3D miniplate group due higher stability of the plate system and easy adaptation. Mohd et al. [27] shown in their study that superior results were obtained with 3D plates, which showed better occlusion, lower infection rates, and less problems with Champy plates. Moreover, Al-Tairi et al. [25] showed no occlusal disturbances among patients treated with 3D miniplate.

The rate of postoperative infection could vary depending on different factors such as the presence of a teeth in the fracture line, as well as the duration between trauma and healing. The lower infection incidence in 3D miniplate studies compared to 2 miniplates could be attributable to the low plate profile, which requires less hardware. Additionally, the 3D plate’s malleability and simultaneous application of upper and lower struts reduced operative time [18, 23–27].

This study has certain limitations. Due to its retrospective design, the data collected for clinical purposes, which may not have been standardized for research. Additionally, the sample size was relatively small, potentially limiting the generalizability of our findings to other populations. Future prospective studies with larger and more diverse patient cohorts are warranted to confirm these results.

Although the current study provides insight about the effect of two miniplates, 2.3 high profile Miniplates and three dimensional (3D) Miniplates for internal fixation treatment of mandibular para-symphyseal fractures but further studies is needed to detect other parameters such as long-term effect of those plate on the underlying bone. We could also recommend a multicenter study to be able to overcome any biases result from analysis of single center data set.

## Conclusion

This study investigated the clinical outcomes of three fixation methods for mandibular fractures: miniplates, reconstruction plates, and 3D miniplates. Our findings revealed no statistically significant differences between these approaches in fracture stability and postoperative complications. These results suggest that all three methods can be effective for achieving successful mandibular fracture management.

In conclusion, miniplate, reconstruction plate and 3D miniplate are effective for management of parasymphyseal fracture. Further research is warranted to explore the potential benefits of 3D-miniplates in a larger patient population and to investigate their long-term durability compared to traditional miniplates and reconstruction plates.

## Data Availability

The datasets used and/or analysed during the current study are available from the corresponding author on reasonable request. For privacy reasons, however, individual data allowing for the identification of participants cannot be made available.

## Abbreviations

MMF: Maxillomandibular fixation
ORIF: Open reduction with internal fixation
IMF: Intermaxillary fixation
IPV: Intimate Partner Violence
IQR: Interquartile range
RTA: Road Traffic Accidents
